# Breath Signatures of Antiseizure Medication Response and Tolerability in Adults With Epilepsy

**DOI:** 10.1002/cns.71169

**Published:** 2026-09-28

**Authors:** Kai Fricke, Diego Manuel Baur, Kapil Dev Singh, Felix Schmidt, Kathrin Müsch, Noriane Andrina Sievi, Marco Di Donato, Anton Schmick, Silvia Ulrich, Pablo Sinues, Marian Galovic, Malcolm Kohler

**Affiliations:** ^1^ Department of Pulmonology University Hospital Zurich Zurich Switzerland; ^2^ Faculty of Medicine University of Zurich Zurich Switzerland; ^3^ University Children's Hospital Basel Basel Switzerland; ^4^ Department of Biomedical Engineering University of Basel Basel Switzerland; ^5^ Department of Neurology University Hospital Zurich Zurich Switzerland

**Keywords:** antiseizure medications, breath metabolomics, epilepsy, pharmacodynamics, treatment responses

## Abstract

**Purpose:**

Achieving seizure control without intolerable adverse effects remains a major challenge in adult epilepsy, and serum concentrations of antiseizure medications correlate poorly with clinical outcomes. Metabolic processes influencing treatment response are not captured by blood monitoring. This study evaluates whether exhaled breath metabolomic profiles reflect treatment response and adverse effects in adults receiving antiseizure therapy, using epilepsy as a model for pharmacodynamic phenotyping.

**Materials and Methods:**

A single‐center observational cohort of 117 adults provided 127 breath samples during stable antiseizure treatment. Exhaled breath samples were analyzed using secondary electrospray ionization high‐resolution mass spectrometry. Machine‐learning models with nested cross‐validation were used to classify treatment response and adverse effects, and pathway‐level analyses were performed to provide biological context. Findings were compared with an independent, predominantly pediatric cohort analyzed using the same workflow.

**Results:**

Breath metabolomic profiles distinguished responders from nonresponders and individuals with and without adverse effects, whereas serum drug concentrations showed no meaningful association. Classification models achieved areas under the curve of 0.84 for treatment response and 0.88 for adverse effects, with good calibration. Pathway‐level alterations were directionally consistent across cohorts.

**Conclusion:**

Breath metabolomics supports a non‐invasive approach to pharmacodynamic phenotyping that captures treatment‐related metabolic states not reflected by serum drug concentrations.

## Graphical abstract

Breath metabolomic profiles distinguish treatment response and adverse effects in adults with epilepsy, whereas serum antiseizure medication concentrations do not. These findings support breath analysis as a non‐invasive approach to pharmacodynamic phenotyping, capturing treatment‐related metabolic states relevant to complex human disease.

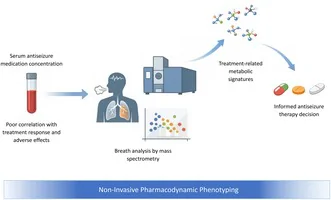

## 1 Introduction

Achieving sustained seizure control without intolerable adverse effects remains a major challenge in epilepsy management [1]. Although antiseizure medications (ASMs) are widely used, subjects vary substantially in their therapeutic response. This variation reflects the influence of multiple metabolic and physiological processes that shape both drug disposition and downstream pharmacodynamic effects [2, 3, 4, 5]. Therapeutic drug monitoring (TDM) provides circulating concentrations but does not capture the broader biochemical activity that contributes to efficacy or intolerance [6]. Serum levels correlate only weakly with clinical outcomes for several widely used agents, highlighting the limited ability of concentration measurements to represent treatment‐relevant biological activity [7, 8, 9]. These processes include mitochondrial and amino acid metabolism, fatty‐acid turnover and pathways involved in neurotransmitter precursor handling, all of which can differ with age, comorbidities, organ function and concomitant medication use [3, 4, 5]. As a result, exposure–response relationships are often inconsistent, and clinicians have limited biomarkers that reflect treatment‐related metabolic activity [8, 9]. Drug response variability in epilepsy therefore provides a clinically relevant model for evaluating whether non‐invasive metabolic phenotyping can capture pharmacodynamic states that are not reflected by circulating drug concentrations.

Metabolic profiling examines these downstream processes directly. Exhaled breath contains volatile end products of systemic metabolism that can indicate shifts in intermediary biochemical pathways relevant to ASM effects. Breath metabolites integrate signals from multiple organ systems, offering a non‐invasive readout of treatment‐related metabolic states rather than drug‐specific mechanisms alone [10]. Real‐time mass‐spectrometric analysis allows repeated sampling and can detect short‐term biochemical fluctuations that may not be apparent in blood‐based measures [11, 12]. Several ASMs influence metabolic processes on relatively rapid timescales. For example, valproate affects mitochondrial function, whereas carbonic‐anhydrase inhibitors alter acid–base balance, suggesting that breath analysis may capture dynamic metabolic patterns associated with treatment response or tolerability [13, 14].

Initial work in a predominantly pediatric cohort identified breath‐derived metabolic patterns associated with seizure control and valproate‐related adverse effects [15]. Whether these signals extend to adult‐only cohorts, characterized by greater clinical heterogeneity, comorbidity, and polypharmacy, remains unknown. These factors may alter metabolic processes in ways that influence both ASM effects and the breath signatures they generate, raising the possibility that treatment‐associated metabolic markers differ between adult and pediatric populations.

In this study, we examined whether breath metabolomic profiles reflect treatment‐related metabolic variation associated with ASM efficacy and tolerability in adults with epilepsy. By relating breath‐derived signatures to well‐defined clinical outcomes, we sought to determine whether non‐invasive metabolic profiling captures biologically relevant patterns of treatment response and may offer markers with potential relevance for individualized management.

## 2 Materials and Methods

### 2.1 Study Design and Participants

We performed a single‐center observational study at the University Hospital Zurich between September 2022 and August 2024. Adults (≥ 18 years) with a confirmed diagnosis of epilepsy and stable treatment with one or more ASMs were eligible. All participants were assessed during routine outpatient visits while in a steady‐state treatment phase. The cohort predominantly comprised patients with established, chronic epilepsy under ongoing specialist care, reflecting long‐term disease management rather than newly diagnosed or unstable cases. Concomitant non‐ASMs were systematically documented as part of the clinical assessment and were further categorized by medication class for descriptive analysis, including cardiovascular, psychotropic, endocrine, and other commonly prescribed drug groups. No formal exclusion criteria were applied based on the use of medications with potential metabolic effects. This approach was chosen to reflect real‐world clinical practice, where polypharmacy and comorbidity are common, and to capture treatment‐related metabolic states under routine conditions. A total of 127 valid breath measurements were obtained from 117 subjects (Figure S1). Ten participants provided repeat measurements at follow‐up visits, resulting in more than one breath sample per individual in these cases.

### 2.2 Clinical Outcome Assessment

Treatment response and ASM‐related side effects were evaluated by board‐certified neurologists during routine clinical assessments. Treatment response was assessed using a structured clinical classification framework based on seizure control and overall clinical course. Responders were defined as patients with favorable clinical response, including seizure freedom or marked reduction in seizure frequency, whereas nonresponders comprised patients with partial or insufficient response [15]. Operational definitions used for classification into response categories are provided in Tables S1 and S2. Side effects were documented through structured patient interviews and physician ratings. Epilepsy etiology was classified according to International League Against Epilepsy (ILAE) criteria. In addition, epilepsy type was categorized as focal, generalized, or unclassified/mixed based on clinical documentation.

### 2.3 Breath Sample Collection and Data Processing

Exhaled breath was collected immediately after venous blood sampling using secondary electrospray ionization coupled to high‐resolution mass spectrometry. To ensure analytical reproducibility and minimize inter‐batch variability, a standardized quality‐control framework was applied throughout the study. Instrument performance was verified at the beginning of each measurement day using a certified reference gas mixture, with acceptance criteria based on multivariate comparison to historical measurements using Hotelling's T^2^ statistics and predefined system suitability thresholds. Internal lock‐mass correction was applied to ensure mass accuracy, and background ion profiles were monitored to detect contamination or carryover. Only measurements acquired after successful completion of quality‐control procedures were included in downstream analyses. Participants provided repeated deep‐lung exhalations under controlled conditions. Raw spectral data were processed using established workflows that included peak detection, alignment, and noise reduction. Features present in fewer than half of samples were excluded, and remaining intensities were log‐transformed. Details of instrumentation, quality‐control procedures, preprocessing, features used, and data‐imputation procedures are described in the Data S1 and Table S4.

### 2.4 Statistical Analysis

Multivariate models were used to examine associations between breath metabolomic profiles and (i) serum ASM concentrations, (ii) treatment response and (iii) adverse effects. Nested cross‐validation was applied to reduce overfitting, and model performance was summarized using standard regression and classification metrics. Within the nested cross‐validation framework, hyperparameters for each model were optimized using an inner cross‐validation loop. Model‐specific parameters, including kernel parameters for support vector machines and the number of components for partial least squares regression, were tuned using structured search procedures. All preprocessing and model tuning steps were performed exclusively within the training data of each fold to prevent information leakage. In addition, repeated measurements from the same participant were assigned to the same cross‐validation folds to avoid information leakage and overrepresentation. Feature rankings were derived through an ensemble of complementary selection techniques, and pathway‐level interpretation was performed using MetaboAnalystR. Sensitivity analyses and full model specifications are provided in the Data S1.

## 3 Results

### 3.1 Breath Metabolomic Profiles Distinguish Treatment Response and Adverse Effects

A total of 117 adults with epilepsy contributed 127 breath samples (Table 1). The cohort comprised a clinically heterogeneous population, with a predominance of focal epilepsy (63%), followed by generalized (24%) and unclassified forms (13%). The distribution of epilepsy types was similar between responders and nonresponders, as well as between individuals with and without adverse effects. Twenty‐eight participants (22%) were classified as nonresponders and 23 (18%) reported clinically significant adverse effects. Nonresponders represented a clinically heterogeneous subgroup, comprising predominantly focal epilepsies with etiologies and ASM regimens similar to those observed in the overall cohort (Table 1). Concomitant non‐ASM medication use was also broadly similar across outcome groups. Overall, 77 of 117 participants (66%) were not receiving any concomitant medication, with comparable proportions among responders (59/89, 66%) and nonresponders (18/28, 64%). Among those receiving additional therapies, drug classes with potential metabolic relevance, including psychotropic agents, endocrine therapies, and analgesics, were infrequent and showed no clear enrichment in nonresponders or in participants reporting adverse effects. Breath‐derived metabolic features differentiated clinical outcome groups, whereas serum ASM concentrations did not. Across all four ASMs, serum levels showed no meaningful association with treatment response or adverse‐effect status (Table S3 and Figure S2).

**TABLE 1:** Cohort characteristics.

	Overall (*n* = 117)	Responders (*n* = 89)	Non‐responders (*n* = 28)	Without side effects (*n* = 94)	With side effects (*n* = 23)
Demographics					
Age, years (SD)	46 (17)	47 (18)	39 (15)	46 (18)	45 (15)
Sex					
Female (%)	35.9	32.6	46.4	34.0	43.5
Male (%)	64.1	67.4	53.6	66.0	56.5
BMI (SD)	24.9 (4.4)	24.8 (4.4)	25.1 (4.2)	24.8 (4.2)	25.3 (5.1)
Clinical variables					
Etiology^a^					
Unknown	31	25	6	29	2
Genetic	25	21	4	22	3
Structural	59	43	16	42	17
Other	2	0	2	1	1
Epilepsy type					
Focal	74	56	18	59	15
Generalized	28	21	7	23	5
Unclassified/mixed	15	12	3	12	3
ASMs					
Valproic acid	25	15	10	18	7
Lamotrigine	67	51	16	54	13
Levetiracetam	33	26	7	24	9
Lacosamide	14	10	4	9	5
Number of ASMs					
1	87	70	17	77	10
2	26	17	9	16	10
3	4	2	2	1	3
4 or more	0	0	0	0	0
Concomitant medications					
Antihypertensives	21	16	5	16	5
Antithrombotic agents	15	11	4	13	2
Lipid‐lowering therapy	7	5	2	5	2
Psychotropic agents	14	11	3	12	2
Analgesics	10	8	2	7	3
Endocrine therapies	8	6	2	7	1
Immunosuppressants	3	2	1	2	1
Other	5	4	1	3	2
None	77	59	18	60	17

*Note:* Values are presented as mean ± SD or *n* (%). Responders were defined as patients who were seizure‐free or achieved the lowest possible seizure frequency for their epilepsy type. Side effects refer to clinically significant antiseizure medication (ASM)–related adverse effects reported at follow‐up. Etiology was classified according to International League Against Epilepsy (ILAE) criteria.

Abbreviations: ASM, antiseizure medication; BMI, body mass index; ILAE, International League Against Epilepsy; SD, standard deviation.

^a^ According to ILAE classification.

Machine‐learning models trained on breath spectra achieved consistent discrimination for both clinical outcomes. The response‐classification model achieved an internally validated AUC of 0.84 (95% CI 0.80–0.88), and the adverse‐effects model achieved an AUC of 0.88 (95% CI 0.85–0.91) (Figure 1). Accuracy, sensitivity, specificity, and likelihood ratios were stable across resampling procedures, and calibration slopes were close to 1. Performance did not improve when demographic variables or serum ASM concentrations were added. Both models demonstrated excellent calibration, with slopes close to 1 and intercepts near 0, indicating that predicted risks closely matched observed event rates. Full performance metrics are provided in Table 2.

**FIGURE 1:**
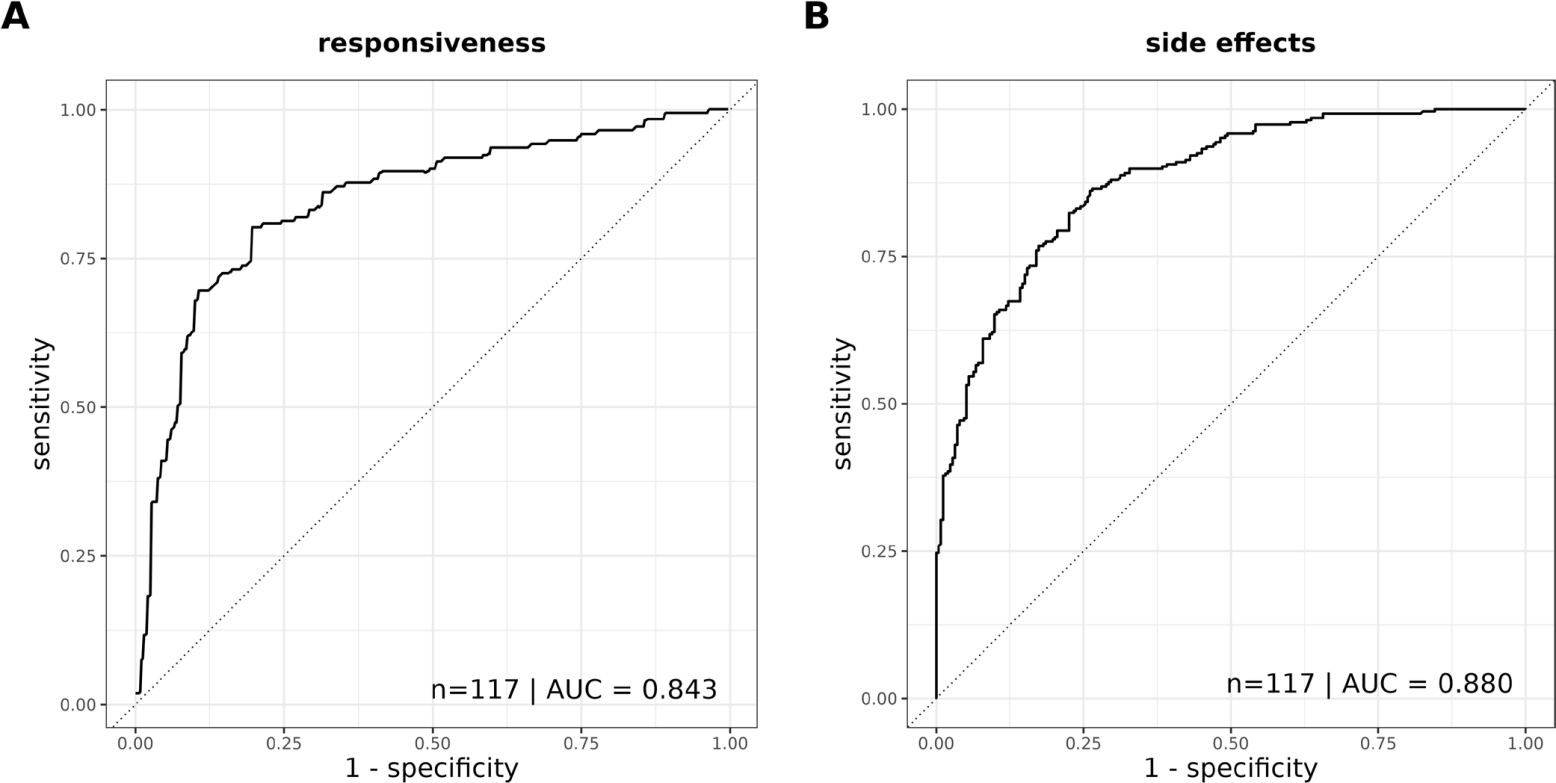
Predictive Performance of Breath‐Based Models for Treatment Response and Adverse Effects in Adults With Epilepsy. Panel A shows the receiver operating characteristic (ROC) curve for the model discriminating treatment responders from non‐responders using breath metabolomic features. Panel B shows the ROC curve for the model distinguishing patients with versus without clinically significant antiseizure medication‐related side effects. Area under the curve (AUC) values with 95% confidence intervals are indicated within each panel.

**TABLE 2:** Performance metrics for breath‐based prediction models of treatment response and side effects.

	Response model	Side effect model
AUC	0.843 (0.798–0.882)	0.880 (0.851–0.905)
Accuracy	0.799 (0.753–0.842)	0.792 (0.758–0.827)
Sensitivity	0.801 (0.747–0.857)	0.794 (0.739–0.843)
Specificity	0.797 (0.739–0.855)	0.791 (0.741–0.839)
PPV	0.792 (0.730–0.851)	0.800 (0.751–0.846)
NPV	0.806 (0.740–0.865)	0.784 (0.734–0.833)
Positive likelihood ratio	3.94 (3.06–5.58)	3.79 (3.03–4.93)
Negative likelihood ratio	0.25 (0.18–0.33)	0.26 (0.20–0.32)
Brier score	0.158 (0.136–0.180)	0.141 (0.124–0.159)
Calibration slope	1.000 (0.783–1.259)	1.000 (0.858–1.178)
Calibration intercept	0.000 (−0.294–0.249)	0.000 (−0.239–0.236)

*Note:* Values are shown with 95% confidence intervals in parentheses.

Abbreviations: ASM, antiseizure medication; AUC, area under the curve; NPV, negative predictive value; PPV, positive predictive value.

### 3.2 Metabolomic Features Associated With Clinical Outcome Groups

Targeted analysis of predefined breath metabolites identified group‐associated metabolic differences (Figure 2). Non‐responders showed lower breath signal intensities for metabolites mapping to the tyrosine and phenylalanine pathways. These included putative annotations corresponding to tyrosine, phenylalanine, dopamine, 3‐methoxytyramine, and homovanillic acid. Two intermediary metabolites, alanine and pyruvate, differed significantly between responders and non‐responders after correction for multiple comparisons (adjusted *p* < 0.05) (Figure S3). Both metabolites were higher in non‐responders. Participants reporting adverse effects showed higher intensities of metabolites associated with amino acid and intermediary metabolism, including alanine, valine, lysine, tryptophan, and phenylalanine.

**FIGURE 2:**
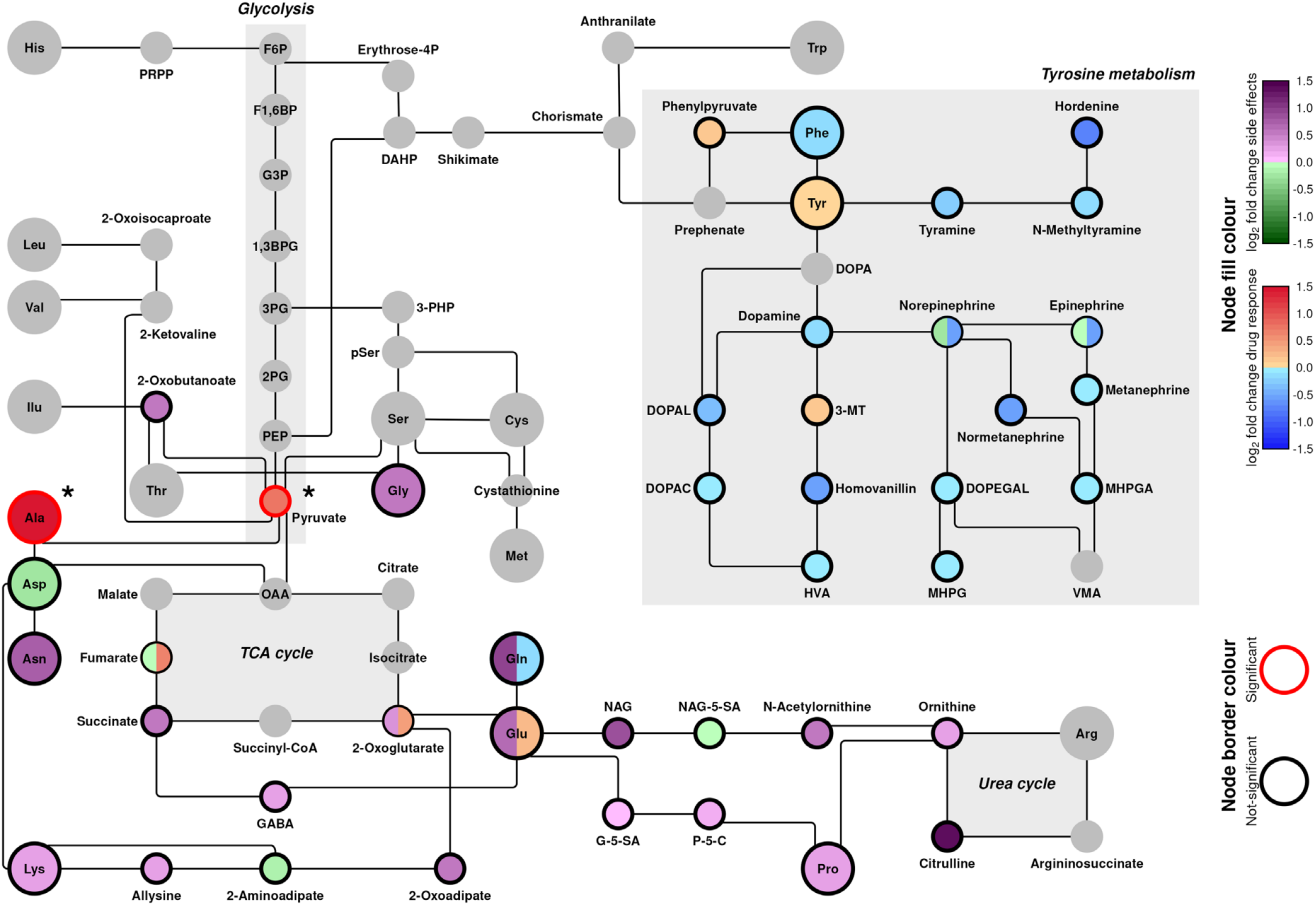
Breath‐based metabolic pathway signatures associated with treatment response and adverse effects in adults with epilepsy. Metabolic pathway context of discriminatory breath metabolites identified by secondary electrospray ionization high‐resolution mass spectrometry (SESI‐HRMS) is shown for treatment response (responders vs. nonresponders) and antiseizure medication–related adverse effects (present vs. absent). Nodes represent putatively annotated metabolites mapped to central metabolic pathways, including amino acid metabolism, glycolysis, the tricarboxylic acid (TCA) cycle, and tyrosine metabolism. Node fill color represents log_2_ fold change between groups, as indicated by the color scale. Node border color denotes statistical significance at the metabolite level. Asterisks (*) indicate metabolites that remained significant after correction for multiple comparisons (adjusted *p* < 0.05). Pyruvate (*p* = 0.044); alanine (*p* = 0.004). Pathway labels provide biological context based on putative metabolite annotations derived from accurate mass matching against the Kyoto Encyclopedia of Genes and Genomes (KEGG) database. Given the untargeted nature of the analysis, metabolite identities and pathway assignments should be interpreted as putative.

### 3.3 Alignment Between Multivariate Modeling and Metabolite‐Level Findings

Features that differed most strongly between clinical groups overlapped with those carrying the highest importance in the predictive models. For treatment response, these included multiple aromatic amino acid pathway members; for adverse‐effect classification, features within amino acid metabolism contributed most prominently. Concordance between univariate and multivariate analyses shows that discrimination arose from structured patterns across metabolites rather than single‐feature fluctuations.

### 3.4 Comparison With Previously Studied Cohorts

The directionality of pathway‐level alterations was consistent with findings from a previously analyzed predominantly pediatric cohort using the same SESI‐HRMS workflow (Figure S4). Despite differences in age distribution, comorbidity burden, and polypharmacy, both cohorts showed lower aromatic amino acid pathway signals in non‐responders and higher amino acid‐related signals in individuals reporting adverse effects.

## 4 Discussion

This study shows that non‐invasive breath metabolomic profiling captures biochemical variation associated with treatment response and adverse effects in adults with epilepsy. Although TDM remains widely used in clinical practice, serum concentrations correlate poorly with efficacy and tolerability for commonly prescribed ASMs. For example, serum levetiracetam concentrations show no meaningful association with seizure control (*p* = 0.616) [8], and a similar disconnect has been reported for lacosamide in a pediatric cohort [9]. Our results extend these observations by demonstrating that breath‐derived metabolic signatures discriminate clinical outcome groups despite comparable serum concentrations, indicating that dynamic metabolic profiling may capture pharmacodynamic activity not reflected by static serum measures.

Across 117 adults, distinct breath metabolic patterns were associated with both ASM‐related adverse effects and treatment non‐response. Two broad pathways emerged: (i) altered aromatic amino acid metabolism, particularly involving the tyrosine–catecholamine axis, and (ii) changes in intermediary energy metabolism reflected in alanine and pyruvate. These pathway‐level alterations provide a biologically plausible link between systemic metabolic states and neuronal excitability. Given that participants were assessed during stable treatment phases and predominantly represented individuals with established, chronic epilepsy, these findings are less likely to reflect acute disease fluctuations and instead may capture more persistent treatment‐related metabolic states. Importantly, the direction of these alterations was consistent with findings from an independently recruited, predominantly pediatric cohort analyzed at a separate center using the same SESI‐HRMS workflow, supporting reproducibility across cohorts. Although exploratory, these findings are consistent with previously documented neurochemical and metabolic mechanisms implicated in both seizure susceptibility and treatment‐resistant epilepsy [16, 17, 18].

Non‐responders showed reduced levels of metabolites mapped to the tyrosine and phenylalanine pathways, including putative features corresponding to tyrosine, phenylalanine, dopamine, 3‐methoxytyramine, and homovanillic acid. These metabolites are central to catecholamine synthesis and degradation. Altered catecholaminergic signaling, particularly within dopaminergic pathways, is known to modulate neuronal excitability through effects on synaptic transmission and cortical–subcortical circuits [19]. Reduced levels may therefore reflect impaired neuromodulatory capacity and contribute to diminished ASM responsiveness. Previous work implicates alterations in monoaminergic signaling pathways, particularly dopaminergic pathways, in the pathophysiology of drug‐resistant epilepsy [20, 21], and reduced activity within the tyrosine–dopamine axis may contribute to impaired ASM responsiveness [22, 23]. These findings suggest broader biochemical inflexibility in neurotransmitter precursor metabolism, which may limit the capacity of neural networks to adapt to ASM–induced modulation of excitability. This interpretation is consistent with transcriptomic analyses showing dysregulation of synaptic and network‐modulatory genes in drug‐refractory temporal lobe epilepsy, as well as network‐level regulomes implicating energy metabolism and neuronal signaling as molecular hallmarks of drug‐resistant epilepsies [24]. Together with metabolomic studies that distinguish individuals who develop pharmacoresistance, these data suggest that impaired metabolic adaptability may accompany poor treatment response.

Alanine and pyruvate were the only metabolites that differed significantly between responders and non‐responders after correction for multiple testing. Both were elevated in non‐responders, indicating a shift in intermediary metabolism linking glycolysis, transamination, and mitochondrial oxidation. This may reflect impaired coupling between glycolysis and oxidative phosphorylation, with consequences for neuronal energy homeostasis. Since several ASMs affect mitochondrial function, these shifts may influence treatment response by altering neuronal bioenergetics. Pyruvate links cytosolic glycolysis with mitochondrial energy production via pyruvate dehydrogenase, whereas alanine reflects activity of alanine aminotransferase within amino acid and nitrogen metabolism [25]. Concurrent elevations may point to altered substrate handling or shifts in redox state, consistent with known metabolic effects of several ASMs [26]. These patterns also resemble metabolic signatures reported in epileptic tissue, including reduced pyruvate dehydrogenase activity, increased reliance on anaerobic metabolism, and mitochondrial inefficiency reported in epileptic tissue from chronic models and surgical resections [27]. Although untargeted metabolomics cannot establish causality, these findings support the hypothesis that subtle perturbations in energy metabolism accompany reduced ASM responsiveness [28].

Participants reporting adverse effects showed increased intensities of metabolites associated with amino acid metabolism, including derivatives of tryptophan, valine, lysine, and phenylalanine. These patterns align with known metabolic effects of ASMs, including alterations in amino acid turnover and mitochondrial function, thereby contributing to neuropsychiatric or systemic adverse effects [29, 30, 31]. Pathway enrichment analysis confirmed over‐representation of amino acid metabolic processes in symptomatic individuals, suggesting that adverse‐effect profiles may reflect broader metabolic responses rather than isolated drug‐specific mechanisms. Amino acid perturbations also provide a biologically plausible link to clinical phenotypes: tryptophan‐derived metabolites affect serotonergic and kynurenine‐pathway signaling [32]; branched‐chain amino acids regulate glutamate–GABA balance [33]; and altered lysine or phenylalanine flux may indicate mitochondrial stress or impaired nitrogen handling, mechanisms relevant to cognitive slowing, mood symptoms and fatigue commonly reported with ASMs [31, 34, 35, 36]. Although effect sizes were modest compared with the previously described pediatric cohort, the concordant direction of pathway‐level alterations suggests that some metabolic responses to ASM exposure may be shared across age groups. The pediatric cohort was independently recruited at a different center and analyzed using the same SESI‐HRMS workflow, enabling cross‐cohort comparison. The similarity in pathway‐level disturbances strengthens biological plausibility and suggests shared biochemical responses to ASM exposure. Although the direction of pathway‐level alterations was consistent across cohorts, differences in the magnitude of metabolic effects may reflect age‐related variation in metabolic regulation. Developmental differences in mitochondrial function, amino acid metabolism, and neurochemical signaling have been shown to influence seizure susceptibility and may contribute to variability in pharmacological response, with pediatric populations often exhibiting greater metabolic plasticity [37, 38]. These factors may modulate the extent to which metabolic pathways are perturbed in response to antiseizure treatment and should be considered when interpreting cross‐cohort comparisons. Such consistency across age groups suggests that these metabolic perturbations may reflect core biochemical pathways engaged by ASM treatment rather than developmental factors alone, a view supported by metabolic and transcriptomic studies reporting impaired plasticity and reduced metabolic resilience in pharmacoresistant epilepsy [18, 39].

Importantly, metabolic features contributing most strongly to outcome discrimination overlapped with those differing between clinical groups, indicating that predictive performance was driven by biologically coherent pathways rather than statistical artifact. The ability of breath profiles to separate responders from non‐responders, when serum concentrations did not, underscores the potential of breath analysis to detect pharmacodynamic processes not captured by traditional TDM. These findings remain exploratory, and the model should be regarded as a proof‐of‐concept rather than a tool ready for clinical deployment. At the same time, the use of standardized acquisition protocols and rigorous quality‐control procedures supports the technical robustness of the breath metabolomic measurements.

In addition to strong discriminatory performance, the breath‐based models demonstrated excellent calibration, indicating that predicted risks closely reflected observed clinical outcomes. The calibration slope was approximately 1.0 in both response and adverse‐effect models, showing that predicted probabilities were well‐scaled and neither systematically inflated nor attenuated. The calibration intercept was near zero, indicating no consistent over‐ or under‐prediction across participants. Moreover, the low Brier scores reflect accurate probability estimates rather than mere binary separation. Together, these metrics suggest that the models captured stable, internally consistent biological signal, lending further confidence to the robustness of the breath‐derived probability estimates despite the modest cohort size. Nevertheless, these calibration findings reflect internal validation only and should not be interpreted as readiness for clinical prediction without external testing.

Although the models yielded robust discrimination, their clinical utility requires careful interpretation. Higher predicted‐risk groups were identified with reasonable positive predictive value, whereas negative predictive value was lower, suggesting that breath analysis currently performs better at identifying individuals at elevated risk of poor outcomes than at excluding risk confidently. These findings highlight the need for larger datasets, ideally spanning multiple centers and ASM classes, to refine calibration and improve generalizability.

Breath analysis offers several potential clinical advantages. The inclusion of patients receiving concomitant medications reflects routine clinical practice and enhances the generalizability of the findings. Concomitant medication use did not appear to drive the observed metabolic differences in this cohort, as two‐thirds of participants were not receiving additional non‐ASM therapy and potentially metabolically relevant drug classes were infrequent and similarly distributed across outcome groups. By capturing endogenous metabolites that reflect integrated biochemical activity across organ systems, breath metabolomics may provide insights into treatment‐related metabolic states not readily accessible through serum concentration measurements [40]. This approach could support early recognition of individuals prone to poor response or adverse effects and guide clinical decisions regarding further evaluation, dose adjustment, or alternative therapies (Figure 3) [41]. Given that breath‐derived metabolites reflect composite physiological states, including hepatic, renal, gastrointestinal, and central nervous system metabolism, this modality may offer a systems‐level view of ASM effects that complements pharmacokinetic monitoring [42]. Because metabolic signatures in breath reflect host physiology rather than drug levels alone, they may complement traditional TDM by adding a pharmacodynamic dimension. However, whether breath markers can predict future clinical trajectories or guide real‐time decision‐making remains unknown.

**FIGURE 3:**
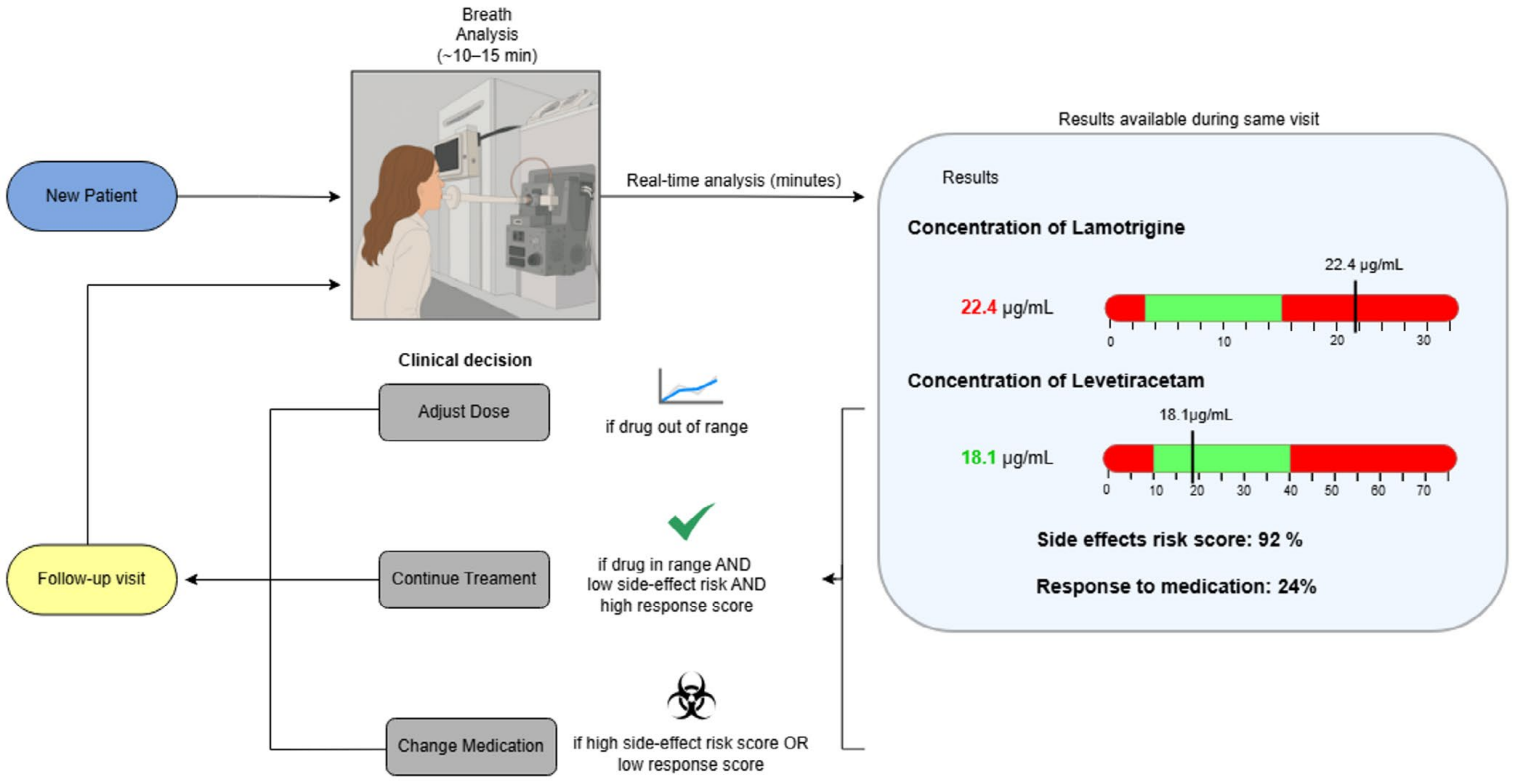
Conceptual clinical workflow for breath metabolomic assessment in epilepsy care. A new patient undergoes SESI–HRMS breath analysis during a routine outpatient visit. Breath acquisition (~10–15 min) is followed by real‐time data processing, enabling generation of metabolomic profiles within the same clinical encounter. The resulting output includes estimated drug exposure, risk of adverse effects, and likelihood of treatment response. These results may support clinical decision‐making, including dose adjustment, continuation of current therapy, or change of antiseizure medication based on integrated risk assessment. The workflow is iterative and may be repeated at follow‐up visits to guide ongoing treatment optimization. The figure illustrates a potential implementation pathway and is intended for conceptual purposes.

Several limitations warrant consideration. First, this was a single‐center study conducted at a tertiary referral center, which may limit generalizability and introduce selection bias. The patient population may not fully reflect broader clinical settings, and external validation in independent, multi‐center cohorts will be required to confirm the robustness and generalizability of these findings. Although analytical procedures were rigorous, including nested cross‐validation and Bayesian imputation, external validation in independent cohorts is required. Model performance may vary due to differences in instrumentation, environmental conditions, or case mix. Second, while several metabolic patterns were biologically plausible and consistent with prior work, pathway‐level associations were modest and did not survive correction in adults, likely reflecting limited power due to relatively small subgroup sizes. In addition, subgroup analyses stratified by individual ASMs were not feasible for outcome‐based comparisons due to limited sample sizes, heterogeneous treatment regimens, and the frequent use of polytherapy. The number of patients with adverse effects was limited (*n* = 23), further restricting the feasibility of stratified analyses. These results should be considered hypothesis‐generating until replicated in larger biomarker‐focused studies. Third, as an observational study, mechanistic causality cannot be inferred; the metabolic differences identified may reflect consequences of disease severity, treatment, or unmeasured confounders. In particular, concomitant non‐ASMs were not explicitly modeled and may have influenced metabolic profiles, especially for agents affecting intermediary or amino acid metabolism. This reflects the real‐world clinical setting but may contribute to residual confounding. However, most participants were not receiving additional non‐ASM therapy, and the distribution of potentially metabolically relevant medication classes was broadly similar across outcome groups, reducing the likelihood that the principal findings were driven by major imbalance in concomitant medication exposure. In addition, correlations between breath‐derived metabolic features and serum drug concentrations were not explored systematically within the scope of the present analysis and are being investigated in dedicated studies. Prospective longitudinal studies are required to assess the temporal stability and predictive capacity of breath metabolomic signatures and to determine whether their integration into clinical decision‐making can improve treatment outcomes. Finally, metabolite annotations from SESI‐HRMS remain putative without tandem‐MS confirmation, and future work should incorporate targeted validation approaches. Detailed information on disease duration was not consistently available for all participants and was therefore not incorporated into the present analysis. However, participants were assessed during stable outpatient follow‐up under ongoing treatment, suggesting that the cohort largely reflects chronic disease states rather than early or rapidly evolving epilepsy. Given that chronicity of epilepsy may influence metabolic states, this represents a potential source of residual heterogeneity. In addition, epilepsy type classification was based on clinical documentation and may not reflect uniform application of formal ILAE criteria across all cases.

Future research should focus on the clinical translation of breath‐based metabolomic profiling into routine epilepsy care. In an outpatient setting, breath analysis offers several potential advantages, including its non‐invasive nature, rapid acquisition time, and the possibility of repeated measurements within a single visit. These features may facilitate dynamic assessment of treatment‐related metabolic states and support more responsive, individualized therapeutic decision‐making. However, several challenges remain for implementation in clinical practice. These include the need for standardization of analytical platforms across centers, validation in larger and more diverse patient populations, and the development of robust, clinically interpretable output metrics. Integration into existing clinical workflows, including turnaround time, cost considerations, and clinician acceptance, will also be critical for successful adoption. Further work should prioritize multi‐center validation, targeted metabolite identification, and prospective trials to determine whether incorporating breath analysis into clinical workflows improves therapeutic outcomes. In particular, prospective interventional studies will be required to assess whether treatment strategies guided by breath‐derived metabolic markers can improve seizure control and reduce adverse effects in clinical practice.

Despite these limitations, our findings support the concept that breath metabolomics captures treatment‐related biochemical variation relevant to ASM efficacy and tolerability. When considered alongside prior pediatric data, these results suggest that breath‐derived metabolic signatures may complement serum‐based TDM by providing additional pharmacodynamic insight into treatment‐related metabolic states. Although not yet ready to replace blood‐based monitoring, this approach offers a non‐invasive, systems‐level perspective on treatment response with potential relevance for individualized epilepsy care.

## Author Contributions

K.F.: conceptualization, methodology, formal analysis and investigation, drafting of the manuscript. D.M.B.: formal analysis and investigation, drafting of the manuscript. K.D.S.: conceptualization, methodology, formal analysis and investigation, critical revision of the manuscript for important intellectual content. F.S.: conceptualization, methodology, formal analysis and investigation, critical revision of the manuscript for important intellectual content. K.M.: formal analysis and investigation, critical revision of the manuscript for important intellectual content. N.A.S.: formal analysis and investigation, critical revision of the manuscript for important intellectual content, formal analysis and investigation. M.D.D.: critical revision of the manuscript for important intellectual content, formal analysis and investigation. A.S.: critical revision of the manuscript for important intellectual content. S.U.: conceptualization, critical revision of the manuscript for important intellectual content. P.S.: conceptualization, methodology, formal analysis and investigation, critical revision of the manuscript for important intellectual content, funding acquisition. M.G.: conceptualization, methodology, critical revision of the manuscript for important intellectual content. M.K.: conceptualization, methodology, critical revision of the manuscript for important intellectual content, funding acquisition.

## Funding

This study was supported by the Lotte and Adolf Hotz‐Sprenger Foundation, the Schwyzer Foundation, the Uniscientia Foundation, and Lunge Zürich. P.S. received funding from Foundation Botnar (Switzerland) and the Swiss National Science Foundation (320030_173168 and PCEGP3_181300).

## Ethics Statement

The study was approved by the Cantonal Ethics Committee of Zurich (University Hospital Zurich, Zurich, Switzerland; BASEC No. 2019–00030), registered at ClinicalTrials.gov (NCT05456009), and conducted in accordance with the Declaration of Helsinki. Written informed consent was obtained from all participants.

## Consent

All participants provided written informed consent.

## Conflicts of Interest

M.K. reports consulting fees from Novartis, Roche Diagnostics, and GSK. M.K. is co‐founder of Deep Breath Intelligence AG, a company that provides services in the field of breath analysis. M.G. received fees from Advisis, Angelini, Bial, Eisai, Neuraxpharm, and UCB outside of the submitted work. P.S. is co‐founder and board observer of Deep Breath Intelligence AG. S.U. receives research grants from the Swiss National Science Foundation, Zurich Lung League, and EMDO foundation and grants, travel support and consultancy fees from Orpha Swiss, Janssen SA, MSD SA, Gebro SA and Ideogen, all unrelated to the present work. K.D.S. and F.S. are part‐time employees of Deep Breath Intelligence AG, a spin‐out from University of Zurich and University of Basel. None declared (K.F., D.M.B., K.M., N.A.S., M.D.D., A.S.).

## Supporting information


**Figure S1:** Flowchart of Breath Measurements and Patient Inclusion.
**Figure S2:** Confusion Matrices Illustrating Model Performance for Predicting Side Effects and Medication Responsiveness across Multiple Outcomes. Classification Performance for Prediction of Clinical Outcome. Each plot represents a normalized confusion matrix as a quadrant plot, with reference values on the vertical axis and predicted outcomes on the horizontal axis. True positives (top left), false positives (top right), false negatives (bottom left), and true negatives (bottom right) are shown as proportions of the total population. Shading intensity corresponds to the magnitude of each cell. Percentages within each quadrant indicate the relative proportion of samples in each classification category.
**Figure S3:** Distribution of alanine‐ and pyruvate‐associated breath metabolite intensities in responders and nonresponders. Box plots show the distribution of log_2_‐transformed normalized intensities of two metabolite features putatively annotated as pyruvate (m/z 87.00877) and alanine (m/z 88.04042) in responders and nonresponders. Each point represents an individual breath sample. Boxes indicate the interquartile range (IQR) with the median, and whiskers represent 1.5× IQR. Both metabolites showed statistically significant differences between groups after correction for multiple testing, with higher intensities observed in responders compared with nonresponders. Corresponding quantitative statistics, including normalized feature importance, log_2_ fold change, raw and adjusted *p*‐values, and annotation details, are provided in Table S4. These box plots provide a visual representation of the group‐level differences underlying the pathway‐level alterations described in the main text, particularly within intermediary energy metabolism and amino acid metabolic pathways.
**Figure S4:** Real‐time breath analysis offers a noninvasive window into altered metabolic pathways in patients not responding to pharmacotherapy and/or suffering from side effects. Adapted from Singh KD, Osswald M, Ziesenitz VC, et al. Personalized therapeutic management of epileptic patients guided by pathway‐driven breath metabolomics. Commun Med. 2021;1 (1):21. Licensed under CC BY 4.0 (https://creativecommons.org/licenses/by/4.0/). Only part A of the original figure (Figure 6) is shown.
**Table S1:** List of side effects used for measurement classification.
**Table S2:** Classification of side effects and response to medication^1^.
**Table S3:** Associations between Antiseizure Medication Levels, Side Effects, and Treatment Response, and Performance of Clinical Prediction Models.
**Data S1:** Detailed description of breath sampling and acquisition, data preprocessing and feature extraction, machine‐learning model development and evaluation, and interpretability and robustness analyses.

**Table S4:** Features used in the prediction models for treatment response and antiseizure medication (ASM)‐related side effects. The corresponding data are provided in a separate Excel file, with Sheet 1 containing features used for treatment‐response prediction and Sheet 2 containing features associated with ASM‐related side effects.

## Data Availability

The data that support the findings of this study are available within the article and its Supporting Information. Table S4 provides the full list of metabolomic features included in the analyses, together with corresponding mass‐to‐charge ratios and annotation information. Additional de‐identified patient‐level data may be made available by the corresponding author upon reasonable request, subject to institutional approvals and applicable ethical and data protection regulations. Detailed descriptions of the analytical workflow, including preprocessing, feature extraction, feature selection, and model development, are provided in the Data S1 to enable reproducibility. The preprocessing pipeline is proprietary; however, all key analytical steps and parameter settings are reported to facilitate independent validation using comparable approaches.
