# Supplementary material for: Breath Signatures of Antiseizure Medication Response and Tolerability in Adults With Epilepsy
Source: CNS Neurosci Ther. 2026 Sep 28;32(10):e71169. doi: 10.1002/cns.71169 (PMC13618557; doi:10.1002/cns.71169)
Supplement: Supplementary file 1 [file CNS-32-e71169-s002.docx]

**Supporting Information**

**Breath Signatures of Antiseizure Medication Response and Tolerability in Adults with Epilepsy**

Kai Fricke, Diego Manuel Baur, Kapil Dev Singh, Felix Schmidt, Kathrin Müsch, Noriane Andrina Sievi, Marco Di Donato, Anton Schmick, Silvia Ulrich, Pablo Sinues, Marian Galovic, Malcolm Kohler

**Table of Contents**

**Supplementary Figure S1**……………………………………………………………...Page 2

Flowchart of participant inclusion and breath sample acquisition

**Supplementary Figure S2**……………………………………………………………...Page 3

Confusion matrices for treatment response and adverse effect classification models

**Supplementary Figure S3**……………………………………………………………...Page 4

Distribution of alanine- and pyruvate-associated breath metabolite intensities in responders and non-responders

**Supplementary Figure S4**……………………………………………………………...Page 5

Real-time breath analysis offers a noninvasive window into altered metabolic pathways in patients not responding to pharmacotherapy and/or suffering from side effects

**Supplementary Methods**……………………………………………………………..Page 6-7

Detailed methods for breath sampling, spectral preprocessing, and machine-learning analysis

**Supplementary Table S1**……………………………………………………………….Page 8

Classification criteria for antiseizure medication–related adverse effects

**Supplementary Table S2**……………………………………………………………….Page 9

Clinical definitions of treatment response and adverse-effect categories

**Supplementary Table S3**…………………………………………………………..Page 10-11

Performance metrics for clinical prediction models

**Supplementary Table S4**……………………………………………………………...Page 11

Features used in the prediction models for treatment response and antiseizure medication (ASM)-related side effects

Attempted breath measurements (n=134)

Excluded (n= 7)

♦  No blood results available (n=3)

♦  Technical issues (n=4)

**Breath measurements included in analysis (n = 127)**

♦  117 unique patients

♦  10 patients underwent a second breath measurement

**Supplementary Figure S1.** Flowchart of Breath Measurements and Patient Inclusion.

USZ training-set

USZ validation set

Side effects

Responsiveness

***Supplementary Figure S2.* Confusion Matrices Illustrating Model Performance for Predicting Side Effects and Medication Responsiveness across Multiple Outcomes.** Classification Performance for Prediction of Clinical Outcome. Each plot represents a normalized confusion matrix as a quadrant plot, with reference values on the vertical axis and predicted outcomes on the horizontal axis. True positives (top left), false positives (top right), false negatives (bottom left), and true negatives (bottom right) are shown as proportions of the total population. Shading intensity corresponds to the magnitude of each cell. Percentages within each quadrant indicate the relative proportion of samples in each classification category.

***Supplementary Figure S3.* Distribution of alanine- and pyruvate-associated breath metabolite intensities in responders and non-responders.** Box plots show the distribution of log₂-transformed normalized intensities of two metabolite features putatively annotated as pyruvate (m/z 87.00877) and alanine (m/z 88.04042) in responders and non-responders. Each point represents an individual breath sample. Boxes indicate the interquartile range (IQR) with the median, and whiskers represent 1.5× IQR. Both metabolites showed statistically significant differences between groups after correction for multiple testing, with higher intensities observed in responders compared with non-responders. Corresponding quantitative statistics, including normalized feature importance, log₂ fold change, raw and adjusted p-values, and annotation details, are provided in Supplementary Table S4. These box plots provide a visual representation of the group-level differences underlying the pathway-level alterations described in the main text, particularly within intermediary energy metabolism and amino acid metabolic pathways.

**Supplementary methods**

***Supplementary Figure S4.* Real-time breath analysis offers a noninvasive window into altered metabolic pathways in patients not responding to pharmacotherapy and/or suffering from side effects. Adapted** from Singh KD, Osswald M, Ziesenitz VC, et al. Personalised therapeutic management of epileptic patients guided by pathway-driven breath metabolomics. Commun Med. 2021;1(1):21. Licensed under CC BY 4.0 (https://creativecommons.org/licenses/by/4.0/). Only part **A** of the original figure (Figure 6) is shown.

SESI–HRMS breath analysis detected alteration in the levels of several amino acids and associated compounds in epileptic patients. The figure shows a simple (unweighted, undirected, no loops, or multiple edges) graph of amino acid metabolism (based on KEGG map01230: biosynthesis of amino acids). Each node is a compound, where node-fill color represents the mean log_2_-scaled fold change in the side effects (yes vs. no), and drug-response (nonresponder vs. responders) dataset from the training set. Node-border color represents whether the compound was assigned to significant or background list via MetaboAnalystR. Only compounds from the significantly enriched pathways (top-right quadrant in Fig. 5) are colored. The rest are shown as grey. Node with two colors (node split) denotes that the compound was present under significantly enriched pathway of both datasets.

***Supplementary Methods.* Detailed description of breath sampling and acquisition, data preprocessing and feature extraction, machine-learning model development and evaluation, and interpretability and robustness analyses.**

**Breath sampling instrumentation and acquisition parameters**

Breath samples were collected using a SUPER-SESI source (Fossil Ion Technology, Spain) coupled to an Orbitrap mass spectrometer (Exactive Plus, Thermo Fisher Scientific, Germany). Measurements were acquired in positive and negative ion modes. Participants exhaled through a sterile filter into a 50 cm SilcoNert-coated stainless-steel tube heated to 130 °C and connected to the curtain AUX gas port of the mass spectrometer. End-tidal CO₂ and exhaled volume were monitored digitally to ensure deep-lung sampling. Each participant provided 12 exhalations (six per mode), with a total sampling duration of ~15 minutes. Breath sampling occurred immediately after venous blood collection (median delay 22 min; IQR 14–35 min). Instrument stability was monitored using daily system suitability testing with a certified reference gas mixture, and analytical performance was evaluated using predefined acceptance criteria. Multivariate comparison of reference measurements was used to detect deviations from expected system behavior. Background ion profiles were assessed to identify potential contamination or carryover.

**Data preprocessing and feature extraction**

Raw spectra were processed using a proprietary pipeline (Deep Breath Intelligence AG, Switzerland). Steps included:

•	peak detection and retention

•	alignment across runs

•	background subtraction and noise filtering

•	removal of features with >50% zero intensities

•	log₁₀-transformation of remaining features

Zeros were treated as left-censored and imputed with a Bayesian lognormal model (PyMC v5.22.0; ArviZ v0.21.0). Convergence was assessed using effective sample size and Gelman–Rubin (R̂) statistics. After preprocessing, 19,790 features were retained (positive mode n = 16,265; negative mode n = 3,525).

**Machine learning workflow**

We implemented a unified modelling pipeline across regression and classification tasks.

**Feature selection**

For each cross-validation fold, ten bootstrap resamples supported robust feature ranking. Importance was estimated independently using:

•	LASSO

•	Boruta

•	minimum redundancy–maximum relevance (mRMR)

•	recursive feature elimination (RFE)

Scores were normalized and aggregated into a consensus ranking.

**Model training and evaluation**

Nested cross-validation was used to minimize overfitting:

•	Outer loop: 10-fold cross-validation repeated ten times

•	Inner loop: 10-fold cross-validation for hyperparameter tuning via tune_race_anova (finetune v1.2.0)

Hyperparameter tuning was performed using predefined search spaces. For support vector machine models, kernel parameters (radial basis function kernel), cost, and sigma were optimized. For partial least squares–based models, the number of latent components was tuned. Optimal parameter combinations were selected based on performance within the inner cross-validation folds.

Serum concentrations without matched breath samples were excluded.

Classification models (random forest, SVM, PLS-DA, LASSO logistic regression) were trained using the same framework. Outcomes were binarized as responder vs non-responder and side-effect vs no side-effect. Performance was summarized using AUC-ROC, accuracy, sensitivity, specificity, PPV, NPV, F₁ score, Cohen’s κ and Matthews correlation coefficient (MCC). To address class imbalance, the majority class was down-sampled within each training iteration.

**Interpretability and robustness assessment**

Model stability was evaluated using 1,000 bootstrap iterations. Medians and 95% confidence intervals were calculated for CCC, AUC, PPV, NPV, RMSE and MAE.

Feature-level interpretability was derived from aggregated importance rankings. Pathway-enrichment analyses were conducted in MetaboAnalystR.

All analyses were performed in R (v4.5.0; tidymodels v1.3.0) and Python (v3.9.21). A two-sided P < 0.05 was considered statistically significant.

***Supplementary Table S1:* List of side effects used for measurement classification**

| Classification | Side effect |
| --- | --- |
| Mild | Drowsiness/fatigue |
|  | Dizziness |
|  | Headache |
|  | Nausea/vomiting |
|  | Tremor |
|  | Weight gain |
|  | Increased appetite |
|  | Sleep disturbances/insomnia |
|  | Visual disturbances |
|  | Mild rash |
|  | Irritability |
|  | Coordination problems/ataxia |
|  | Alopecia |
| Severe | Hepatotoxicity |
|  | Pancreatitis |
|  | Hyperammonemia/encephalopathy |
|  | Thrombocytopenia |
|  | Severe skin reactions |
|  | DRESS syndrome |
|  | Aseptic meningitis |
|  | Multiorgan hypersensitivity |
|  | Psychiatric/behavioral issues (aggression, psychosis, depression) |
|  | Severe mood changes/suicidal ideation |
|  | PR interval prolongation/arrhythmia |
|  | Syncope |

***Supplementary Table S2:* Classification of side effects and response to medication^[1]^**

|  | Category | |
| --- | --- | --- |
| Class | Side effects | Drug response |
| I | No side effects | No seizures or least possible number of seizures for the type of epilepsy |
| II | Mild side effects | Reduction in the number of seizures by more than or at least 50%^1^ |
| III | Several mild or at least one severe side effect | Reduction in the number of seizures by less than 50%^1^ |
| ^1^ compared to phase of highest seizure activity | | |

***Supplementary Table S3.* Associations between Antiseizure Medication Levels, Side Effects, and Treatment Response, and Performance of Clinical Prediction Models.**

**A. Chi-Squared Test Results: Side Effects vs. Therapeutic Range**

| *Medication* | *p-value* |
| --- | --- |
| Total valproic acid | 0.726 |
| Free valproic acid | 1.000 |
| Levetiracetam | 0.669 |
| Lamotrigine | 0.317 |
| Lacosamide | 0.765 |

**B. Machine Learning Performance: Prediction of Side Effects Using Clinical Variables (age, sex, BMI, number of ASMs)**

| *Model* | *Accuracy* | *Sensitivity* | *Specificity* | *ppv* | *npv* | *f1* | *kap* | *mcc* | *roc_auc* |
| --- | --- | --- | --- | --- | --- | --- | --- | --- | --- |
| K-Nearest Neighbors | 0.622 | 0.543 | 0.718 | 0.699 | 0.566 | 0.611 | 0.255 | 0.262 | 0.627 |
| Logistic Regression | 0.567 | 0.527 | 0.613 | 0.605 | 0.535 | 0.563 | 0.139 | 0.14 | 0.586 |
| Support Vector Machine | 0.56 | 0.453 | 0.692 | 0.644 | 0.507 | 0.532 | 0.141 | 0.148 | 0.584 |
| Naive Bayes | 0.571 | 0.406 | 0.755 | 0.65 | 0.532 | 0.5 | 0.158 | 0.171 | 0.553 |
| Random Forest | 0.554 | 0.509 | 0.605 | 0.591 | 0.524 | 0.547 | 0.113 | 0.114 | 0.545 |
| Neural Network | 0.515 | 0.446 | 0.593 | 0.552 | 0.488 | 0.494 | 0.039 | 0.04 | 0.53 |
| Decision Tree | 0.59 | 0.509 | 0.68 | 0.64 | 0.553 | 0.567 | 0.187 | 0.191 | 0.511 |

**C. Logistic regression predicting side effects via drug concentration:**

p-value of drug concentration

0.412

**D. Chi-Squared Test Results: Responsiveness**

| *Medication* | *p-value* |
| --- | --- |
| Total valproic acid | 0.134 |
| Free valproic acid | 0.358 |
| Levetiracetam | 1.000 |
| Lamotrigine | 0.647 |
| Lacosamide | 0.596 |

**E. Machine Learning Performance: Prediction of Responsiveness Using Clinical Variables (age, sex, BMI, number of ASMs).**

| *Model* | *Accuracy* | *Sensitivity* | *Specificity* | ppv | npv | f1 | kap | mcc | roc_auc |
| --- | --- | --- | --- | --- | --- | --- | --- | --- | --- |
| Logistic Regression | 0.579 | 0.521 | 0.636 | 0.584 | 0.575 | 0.55 | 0.157 | 0.158 | 0.644 |
| Neural Network | 0.571 | 0.517 | 0.625 | 0.575 | 0.569 | 0.544 | 0.142 | 0.143 | 0.61 |
| Random Forest | 0.556 | 0.476 | 0.634 | 0.559 | 0.553 | 0.514 | 0.11 | 0.111 | 0.569 |
| Naive Bayes | 0.565 | 0.315 | 0.81 | 0.618 | 0.547 | 0.417 | 0.125 | 0.143 | 0.548 |
| Decision Tree | 0.546 | 0.483 | 0.608 | 0.547 | 0.546 | 0.513 | 0.091 | 0.092 | 0.546 |
| K-Nearest Neighbors | 0.516 | 0.406 | 0.629 | 0.531 | 0.505 | 0.46 | 0.035 | 0.036 | 0.52 |
| Support Vector Machine | 0.476 | 0.373 | 0.585 | 0.486 | 0.47 | 0.422 | -0.042 | -0.043 | 0.466 |

**F. Logistic regression predicting responder class via drug concentration**

p-value of drug concentration

0.273

***Supplementary Table S4.* Features used in the prediction models for treatment response and antiseizure medication (ASM)-related side effects.** The corresponding data are provided in a separate Excel file, with Sheet 1 containing features used for treatment-response prediction and Sheet 2 containing features associated with ASM-related side effects.

1. as described previously: Singh KD, Osswald M, Ziesenitz VC, Awchi M, Usemann J, Imbach LL, Kohler M, García-Gómez D, van den Anker J, Frey U, Datta AN. Personalised therapeutic management of epileptic patients guided by pathway-driven breath metabolomics. Communications medicine. 2021 Aug 2;1(1):21.
